# Comparison of programmed intermittent epidural bolus injection and continuous epidural injection in controlling nighttime pain and improving sleep quality after thoracotomy

**DOI:** 10.1097/MD.0000000000031684

**Published:** 2022-11-11

**Authors:** Su-Sung Lee, Ji-Hye Baek, Soon-Ji Park, Hye-Jin Kim, Hee-Young Kim, Gyeong-Jo Byeon

**Affiliations:** a Department of Anesthesia and Pain Medicine, Pusan National University Yangsan Hospital, Pusan National University School of Medicine, Busan, South Korea; b Research Institute for Convergence of Biomedical Science and Technology, Pusan National University Yangsan Hospital, Yangsan, South Korea.

**Keywords:** epidural analgesia, programmed intermittent bolus, sleep quality, thoracotomy

## Abstract

Postoperative pain after open thoracotomy is known to be very severe and affects sleep quality. This study aimed to investigate the effects of a programmed intermittent epidural bolus injection versus continuous epidural injection for controlling nighttime pain and improving sleep quality after thoracotomy.

**Methods:**

Seventy-six patients scheduled for open thoracotomy for lung cancer or other lung diseases were enrolled. The participants were divided into 2 groups. Group A was continuously injected with 0.2% levobupivacaine at 1.1 mL/h, and group B was injected intermittently with 3 mL 0.2% levobupivacaine at 3 hours intervals through a thoracic epidural catheter via a programmed infusion pump. Within 48 hours after surgery, the degree of pain control using visual analog scale and the patients’ sleep conditions on postoperative day (POD) 0 and 1 were evaluated, and other adverse events were investigated.

**Results:**

On POD 1 night, the visual analog scale in group B showed lower than group A (*P* = .009). Comparison of time to fall asleep showed no differences between 2 groups. Total sleep time was no difference on POD 0 but was longer in group B than that in group A on POD 1 (*P* = .042). Awakening from sleep on POD 0 was lower in group B than that in group A (*P* = .033), and satisfaction with sleep quality on POD 0 was superior in group B compared to group A (*P* = .005). Postoperative nausea and vomiting occurred more frequently in group B than in group A (*P* = .018).

**Conclusion:**

The programmed intermittent epidural bolus technique of patient-controlled epidural analgesia reduces postoperative nighttime pain and improves sleep quality in patients undergoing thoracotomy for lung cancer or other lung diseases.

## 1. Introduction

Postoperative pain in patients undergoing open thoracotomy for lung cancer or other lung diseases is severe. In particular, recovery of spontaneous breathing after awakening from anesthesia is difficult immediately after thoracotomy.^[1]^ In addition, when nighttime breakthrough pain occurs after surgery, the patient requires additional analgesia, and the quality of sleep worsens.^[2]^ If pain control is not satisfied during the nighttime, sleep disturbance will occur, which then negatively affects recovery. Therefore, proper pain control after open thoracotomy is important, and it is known that pain control using epidural analgesia is the most effective method.^[3]^

Several studies have shown that the programmed intermittent epidural bolus (PIEB) injection is effective in epidural autologous pain control devices for labor pain.^[4,5]^ Furthermore, studies on the effects of open or laparoscopic abdominal surgery are being actively conducted.^[6–8]^ However, few cases of using PIEB injection in thoracotomy, and a study reported that using PIEB injection reduced the use of local anesthetics.^[8]^

While previous studies have mainly investigated the pain control effect of the PIEB injection, this study hypothesized that using the PIEB injection at night would allow the patient to get good quality sleep without waking up to press the bolus of the patient-controlled epidural analgesia (PCEA) device for pain. Therefore, our study compared PIEB injection and continuous epidural injection using a PCEA device for nighttime pain control after thoracotomy.

The present study aimed to investigate the effects of nighttime pain control after thoracotomy according to the administration method as the primary outcome and to examine the effects of these pain controls on sleep improvement and postoperative recovery of patients as secondary outcomes.

## 2. Methods

### 2.1. Patient enrollment

The study protocol was approved by the Institutional Review Board of Pusan National University Yangsan Hospital (ID 05–2020-115) and registered with the Clinical Research Information Service (registration number: KCT0005198, https://cris.nih.go.kr/cris/search/detailSearch.do/). After obtaining written informed consent, we enrolled patients aged > 19 years with American Society of Anesthesiologists physical status I–III scheduled for open thoracotomy for lung cancer or other lung diseases. Only the first scheduled surgery in the morning was performed to match the post-surgery investigation time. Patients with neurological or intellectual disabilities, spinal deformities, blood coagulation disorders, neurologic defects at the site, sleep disorders, or patients taking sedatives or sleeping pills, or patients who had allergic reactions to levobupivacaine in previous surgeries, were excluded from the study.

### 2.2. Randomization

At the preanesthetic visit, all patients were fully informed of how to use a patient-controlled analgesic device, the randomization protocol, and pain assessment using the visual analog scale (VAS; with 0 being “no pain” and 10 being “the worst pain imaginable”), before being accepted into the study. Randomization of patient into 2 groups were done using a list of random numbers generated program of Microsoft Office Excel (Microsoft Corp., Redmond, WA). The participants were divided into the control group (group A in which 0.2% levobupivacaine was continuously injected at 1.1 mL/h) and an experimental group (group B, a group injected with 3 mL of 0.2% levobupivacaine intermittently at 3 h intervals).

### 2.3. Procedure

An epidural catheter was inserted into T6–T7, or T7–T8 levels for PCEA. As a test dose, 60 mg lidocaine and 15 μg epinephrine were injected through the catheter, and after confirming no hemodynamic change or neurological change, general anesthesia induction was performed. General anesthesia was induced by intravenous administration of 2 mg/kg propofol, and 0.5–1.0 μg/kg/min remifentanil, then the conscious loss was identified, and 0.8 mg/kg rocuronium was administered to obtain muscle relaxation. The train-of-four count was 0, and intubation was performed using a dual-lumen endotracheal tube. After performing the intubation and stabilizing the hemodynamic state, a volume of 7.5 mL 0.2% levobupivacaine (15 mg levobupivacaine + 50 μg fentanyl) was administered as a loading dose through the epidural catheter. For thoracotomy, anesthesia was maintained with 2 vol% sevoflurane, and 0.2 mg/kg rocuronium was administered intraoperatively at 40 to 60 minutes intervals for muscle relaxation.

After the surgery was completed, a total volume of 150 mL 0.2% levobupivacaine (levobupivacaine 300 mg + fentanyl 500 μg) was infused through the epidural catheter via a portable electronic infusion pump (Accumate 1100®; Woo Young Medical Co., Ltd., Jincheon, Chungbuk, Korea). In group A, the infusion pump was programmed such that 0.2% levobupivacaine was continuously injected at 1.1 mL/h; if the patients felt pain, further infusion of 3 mL of 0.2% levobupivacaine when the patient wanted an additional analgesic, and the lock-out time was 30 minutes. In group B, the infusion pump was programmed with 3 mL of 0.2% levobupivacaine periodically at 3 hours intervals, continuously injected at 0.1 mL/h, and a patient-requiring bolus dose of 3 mL with a lockout time of 30 minutes through the catheter, similar to group A. If the patients did not press the additional patient’s requirement button, both groups were set to infuse the same amount of local anesthetic. When the VAS score was > 4 and the patient wanted additional analgesics during the postoperative period, 30 mg ketorolac was injected. If pain with a VAS score > 4 persisted, 25–50 mg of pethidine was administered.

### 2.4. Outcome measurements

An investigator blinded to the group assignments assessed study outcomes. The VAS was recorded when the patient could communicate voluntarily after admission in the postanesthetic care unit (PACU), and on postoperative day (POD) 0 night (between 9 p.m. and 11 p.m.), POD 1 day (between 9 a.m. and 11 a.m.) and night, and POD 2 day. Additional analgesic requirements within 48 hours after surgery were documented as the incidence and usage of patients requiring additional analgesics by the investigator.

To evaluate the patients’ sleep conditions, the time to fall asleep, total sleep time, number of times awakened from sleep, and quality of sleep were evaluated. The satisfaction of sleep quality was assessed using a 5-point scale,^[9]^ with 5 = very satisfied, 4 = satisfied, 3 = neutral, 2 = dissatisfied, and 1 = very dissatisfied. The investigator asked the patients if they woke up during sleep for reasons such as “to go to the bathroom,” “dyspnea,” “pain,” “anxiety,” and “noise” and recorded the incidences.

Adverse events, including postoperative nausea and vomiting (PONV), urinary retention, dizziness, and paresthesia, were also evaluated. Patient satisfaction for postoperative pain management was assessed using a 5-point scale, same as above.

After the administration of the drug through the patient-controlled device, the portable electronic injection pump was collected and connected to the computer to analyze the pump usage patterns of the subjects for 48 hours after surgery. The definition of each usage pattern is as follows: total dose, total dose of local anesthetic administered for 48 hours after turning on the portable electronic injection pump; nightly dose, local anesthetic dose used from 10 p.m. to 6 a.m. the next day; frequency of bolus injection, frequency of bolus use in 48 hours after switching on the portable electronic injection pump; frequency of using bolus at night, frequency of bolus use from 10 pm to 6 am the next day. The analysis program used Acculinker, version 1.0 (Woo Young Medical Co., Ltd., Jincheon, Chungbuk, Korea).

### 2.5. Statistical analysis

Statistical analysis was performed using IBM SPSS Statistics for Windows, version 27.0, (IBM Corp., Armonk, NY). For the demographic data, a Student’s *t* test was used for the numerical data, and the Chi-squared test was used for categorical data. A *t* test was used to compare the VAS score and total dose of local anesthetics. The incidences of adverse events, patient satisfaction with postoperative pain management, times of bolus injection using a local anesthetic delivery injection pump, and rescue analgesic administration were compared using the Chi-squared test or Fisher’s exact test. Statistical significance was set at *P* < .05.

### 2.6. Sample size estimation

This study compared post-thoracotomy pain during the nighttime between 2 groups. The 2 groups assumed a clinically significant difference when the average difference in VAS measured after 12 hours of surgery was 1.5 or more. As the VAS was measured at 6.5 ± 2.2 in a previous study^[6]^, the sample size was calculated with a standard deviation of 2.2 and a type I (α) and type II (β) error of 0.05 and 0.2, respectively. A total of 76 patients were recruited, of which 38 were assigned to each group.

## 3. Results

**Figure 1.**
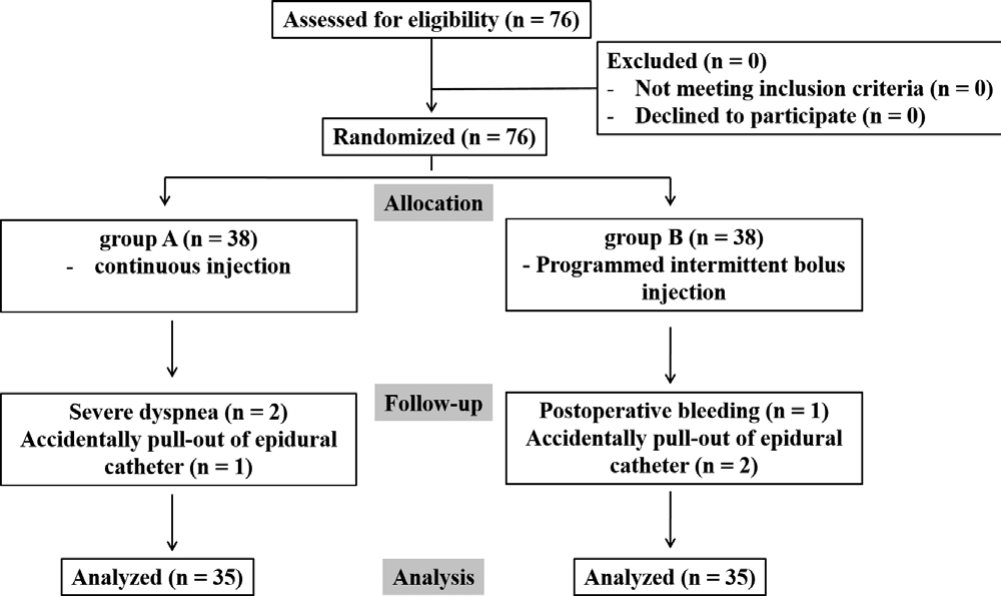
Patient enrollment and a study flowchart.

This study included 76 patients. There were 3 eliminated patients in both groups. Two patients in group A stopped infusion into the epidural catheter due to breathing difficulties, and one patient dislodged the epidural catheter unexpectedly. One patient in group B was hospitalized in intensive care for a long period of time after surgery due to intrathoracic surgical site bleeding, and 2 patients dislodged the epidural catheter unexpectedly. Two patients who were stopped due to breathing difficulties and 3 patients who dislodged the epidural catheter were replaced by opioid-based intravenous patient-controlled analgesia (Fig. 1). There were no differences in demographic data between 2 groups (Table 1).

**Table 1:** Demographic data.

Characteristics	Group A (n = 35)	Group B (n = 35)	*P* value
Sex (M/F)	25/10	23/12	.797
ASA physical status (I/II/III)	13/20/2	10/22/3	.709
Age (yr)	64.1 ± 7.9	62.1 ± 10.2	.264
Height (cm)	162.7 ± 9.5	164.5 ± 7.4	.324
Weight (kg)	63.1 ± 11.9	62.2 ± 10.8	.910
Anesthesia time (h)	4.3 ± 1.3	4.6 ± 1.3	.861

All measured values are presented as number of patients or mean ± standard deviation. Group A, a group in which 0.2% levobupivacaine was continuously injected at 1.1 mL/h; Group B, a group injected with 3 mL of 0.2% levobupivacaine intermittently at 3 h intervals.

ASA = American Society of Anesthesiologists.

**Figure 2.**
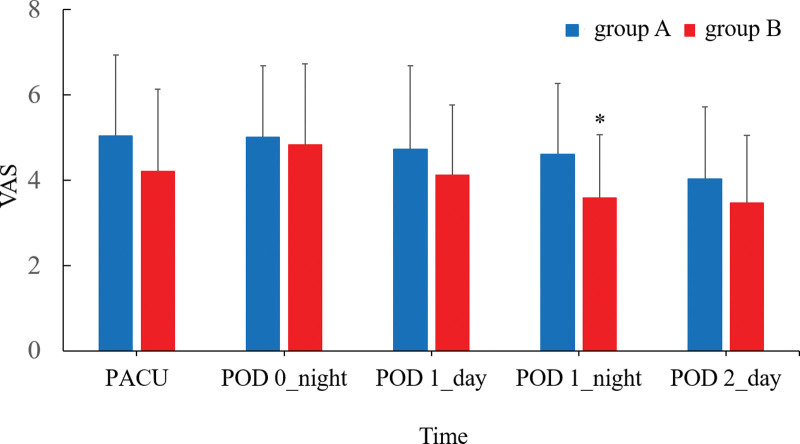
The VAS scores when the patient can communicate voluntarily after admission to the PACU, at POD 0 night, POD 1 day and night, and POD 2 day. On the POD 1 night, Group B showed statistically significantly lower VAS than Group A (*P* < .05). * *P* < .05; PACU, postanesthetic care unit; POD, postoperative day; POD 0 night, from 11 p.m. on the day of surgery to 9 a.m. the next day; POD 1 day, 9 a.m. to 11 p.m. the day after surgery; Group A, a group in which 0.2% levobupivacaine was continuously injected at 1.1 mL/h; Group B, a group injected with 3 mL of 0.2% levobupivacaine intermittently at 3 h intervals. PACU = postanesthetic care unit, POD = postoperative day, VAS = visual analog scale.

The VAS when the patient could communicate voluntarily after admission to the PACU, at POD 0 night, POD 1 day and night, and POD 2 day were shown in Fig. 2. The mean differences (95% confidence interval) of the postoperative VAS when the patient could communicate voluntarily after admission to the PACU, at POD 0 night, POD 1 day and night, and POD 2 day were 0.82 (−0.09 to 1.74), 0.17 (−0.68 to 1.02), 0.61 (−0.25 to 1.47), 1.01 (0.27 to 1.76), and 0.57 (−0.22 to 1.34), respectively. On POD 1 night, group B showed significantly lower VAS than group A (*P* = .009, Fig. 2). The incidence of patients requiring additional analgesics within 48 hours after the operation showed no difference between 2 groups. After administration of the drug through the patient-controlled device, the portable electronic injection pump was collected and connected to the computer to analyze the pump usage patterns (total dose, nightly dose, frequency of bolus injection, and frequency of bolus use at night) of the patients for 48 hours after surgery. When comparing the administration of the drug through the patient-controlled device in the 2 groups, there was no difference in the total dose, nightly dose, frequency of bolus injection, and frequency of using bolus at night (Table 2).

**Table 2:** The incidence of patients requiring additional analgesics and administration of the drug through the portable electronic injection pump within 48 h after surgery.

Incidence of patients requiring additional analgesics	Group A (n = 35)	Group B (n = 35)	*P* value
PACU	8 (22.9)	10 (28.6)	.584
POD 0 night	13 (37.1)	16 (45.7)	.467
POD 1 day	14 (40.0)	13 (37.1)	.806
POD 1 night	18 (51.4)	13 (37.1)	.229
POD 2 day	14 (40.0)	8 (22.9)	.122
Administration of the drug through the portable electronic injection pump			
Total dose (mL)	105.1 ± 24.0	103.7 ± 24.9	.811
Nightly dose (mL)	31.9 ± 13.2	28.1 ± 8.2	.149
Frequency of bolus injection	19.5 ± 10.8	16.3 ± 8.3	.176
Frequency of using bolus at night			
POD 0_night	3.7 ± 2.9	2.7 ± 2.2	.123
POD 1_night	2.5 ± 2.2	1.7 ± 1.5	.064

All measured values are presented as mean ± standard deviation or number of patients (%). Group A, a group in which 0.2% levobupivacaine was continuously injected at 1.1 mL/h; Group B, a group injected with 3 mL of 0.2% levobupivacaine intermittently at 3 h intervals. POD 0 night, from 11 p.m. on the day of surgery to 9 a.m. the next day; POD 1 day, 9 a.m. to 11 p.m. the day after surgery; VAS, visual analog scale; Total dose, total dose of local anesthetic administered for 48 h after turning on the portable electronic injection pump; Nightly dose, local anesthetic dose used from 10 pm to 6 am the next day; Frequency of bolus injection, frequency of bolus use in 48 hours after switching on the portable electronic injection pump; Frequency of using bolus at night, frequency of bolus use from 10 pm to 6 am the next day.

PACU = postanesthetic care unit, POD = postoperative day.

The comparison of sleep conditions between the 2 groups showed no difference in the time to fall asleep and total sleep time. On the other hand, awakened from sleep showed a lower incidence in group B than in group A, and the quality of sleep was also more satisfactory for group B on POD 0 night (*P* = .005). On POD 1 night, the time to fall asleep did not make a difference, but the total sleep time was longer in group B (*P* = .042). When the researcher asked patients about the reason they woke up during the night, the percentage of respondents who said they woke up due to pain was higher in group A than in group B on POD 0 night (*P* = .002, Table 3).

**Table 3:** Patients’ sleep conditions.

			Group A (n = 35)	Group B (n = 35)	*P* value
POD 0	Time to fall asleep (min)		21.7 ± 16.2	17.7 ± 15.0	.287
	Total sleep time (h)		5.3 ± 1.8	5.6 ± 2.1	.502
	Awakened from sleep (times)		4.6 ± 3.1	3.1 ± 2.9*	.033
	Satisfaction with sleep quality	5 = very satisfied	4 (11.4)	9 (25.7)	.005
		4 = satisfied	6 (17.1)	16 (45.7)*	
		3 = neutral	21 (60.0)	8 (22.9)	
		2 = dissatisfied	4 (11.4)	2 (5.7)	
		1 = very dissatisfied	0 (0.0)	0 (0.0)	
	Reason for waking up	To go to the bathroom	2 (5.7)	1 (2.9)	1.000
		Dyspnea	7 (20.0)	4 (11.4)	.513
		Pain	22 (62.9)	9 (25.7)*	.002
		Anxiety	3 (8.6)	7 (20.0)	.306
		Noise	2 (5.7)	7 (20.0)	.151
POD 1	Time to fall asleep (min)		14.7 ± 9.8	14.9 ± 12.9	.959
	Total sleep time (h)		6.2 ± 1.3	6.8 ± 1.2*	.042
	Awakened from sleep (times)		2.5 ± 2.3	1.7 ± 1.9	.146
	Satisfaction with sleep quality	5 = very satisfied	7 (20.0)	17 (48.6)	.064
		4 = satisfied	18 (51.4)	14 (40.0)	
		3 = neutral	8 (22.9)	3 (8.6)	
		2 = dissatisfied	2 (5.7)	1 (2.9)	
		1 = very dissatisfied	0 (0.0)	0 (0.0)	
	Reason for waking up	To go to the bathroom	6 (17.1)	3 (8.6)	.477
		Dyspnea	5 (14.3)	4 (11.4)	1.000
		Pain	17 (48.6)	10 (28.6)	.086
		Anxiety	2 (5.7)	3 (8.6)	1.000
		Noise	2 (5.7)	6 (17.1)	.259

All measured values are presented as mean ± standard deviation or number of patients (%). Group A, a group in which 0.2% levobupivacaine was continuously injected at 1.1 mL/h; Group B, a group injected with 3 mL of 0.2% levobupivacaine intermittently at 3 h intervals. POD = postoperative day.

* *P* < .05.

Adverse events, including PONV, urinary retention, dizziness, and paresthesia were evaluated. Other adverse events did not show a difference between 2 groups, but PONV occurred more frequently in group B than in group A (*P* = .018, Table 4). There was no significant difference in Patient satisfaction for postoperative pain management between 2 groups.

**Table 4:** Adverse events.

Adverse events	Group A (n = 35)	Group B (n = 35)	*P* value
Postoperative nausea and vomiting	3 (8.6)	12 (34.3)*	.018
Urinary retention	2 (5.7)	2 (5.7)	1.000
Dizziness	4 (11.4)	6 (17.1)	.734
Paresthesia	0 (0.0)	1 (2.9)	1.000
Pruritus	1 (2.9)	1 (2.9)	1.000
Headache	1 (2.9)	0 (0.0)	1.000
Delirium	1 (2.9)	0 (0.0)	1.000
Back pain	0 (0.0)	1 (2.9)	1.000
Dyspnea	1 (2.9)	0 (0.0)	1.000
Hiccup	0 (0.0)	1 (2.9)	1.000

All measured values are presented as number of patients (%). Group A, a group in which 0.2% levobupivacaine was continuously injected at 1.1 mL/h; Group B, a group injected with 3 mL of 0.2% levobupivacaine intermittently at 3 h intervals.

* *P* < .05.

## 4. Discussion

This study was conducted to investigate the effects on patients through various observations of postoperative pain management when applying the PIEB injection with a patient-controlled analgesia device in patients who underwent open thoracotomy. Although postoperative pain management through epidural injection has already been proven to be effective, the results of each difference and the pros and cons of drug injection techniques remain unknown. However, several recent studies on PCEA have shown that the PIEB injection of the PCEA reduces motor block and shows a better analgesic effect.^[10–12]^

A study has been demonstrated that the use of intermittent epidural bolus injection may result in a more extensive spread of local anesthetic in the epidural space due to the pressure effect. Furthermore, it is associated with a reduced dose of local anesthetic and better patient satisfaction than continuous epidural infusion.^[13]^ Meanwhile, the other study showed the limitation of continuous infusion of epidural technique, which is ununiformed spreading with inked injection to cadavers.^[14]^

In this study, the PIEB injection showed less pain at POD 1 night compared to the continuous epidural injection. However, there was no difference in the incidence of patients requiring additional analgesics within 48 hours after surgery when postoperative pain control was not satisfactory between 2 injections. When comparing the administration of the drug through the PCEA device in the 2 injections, the total consumption of local anesthetics and frequency of bolus injection in case of breakthrough pain did not show significant differences. Regarding the incidence of adverse events, the PIEB injection had a higher incidence of PONV. The results of this study were similar to those of previous studies but also showed contradicting results. In major abdominal and gynecological cancer surgery, the PIEB injection had fewer bolus uses, but there were no significant results in other outcomes, including additional morphine requirements and pain scores.^[12]^ In the control of labor pain, a study showed that the PIEB injection had a lower rate of breakthrough pain and reduced the use of additional analgesics compared to the continuous epidural injection,^[15]^ whereas another study showed that the PIEB injection had less motor block, but there were no significant results in other outcomes.^[16]^ In gynecologic surgery, some studies showed that the PIEB injection used less total local anesthetic for 40 h after surgery and lower postoperative pain intensity.^[7]^ In thoracic surgery, the PIEB injection reduced the amount of local anesthetic, but the frequency of adverse events, such as hypotension, increased.^[8]^

Postoperative sleep disturbance is a problem that occurs frequently to the patients, and it is an element that can adversely affect prognostic cases in severe cases.^[2]^ There are various factors that affect the decline in sleep quality, but the greater the number of major surgeries, the greater the contribution of postoperative pain. Therefore, open thoracotomy with severe pain is likely to have a quality decline in sleep, and paradoxically, it can be an evaluation factor for pain control. In this study, local anesthetics were administered through an epidural catheter at intervals of 3 hours in the program using the PIEB injection to prevent patients from waking up due to pain during the night after the operation. It is assumed that local anesthetics are administered at a certain time before the patient wakes up in pain, which will improve the quality of sleep. Our research findings showed that the use of the PIEB injection resulted in less waking up on POD 0 due to pain, resulting in more satisfactory quality of sleep.

In previous studies, if the PIEB injection was mainly evaluated for postoperative pain improvement, our study included not only pain improvement but also quality of sleep as an evaluation item, which is meaningful in that postoperative pain was evaluated in multiple ways, not just by scoring it.

Among the adverse events in this study, PONV was higher in patients who underwent the PIEB technique. Other studies using the PIEB technique have reported no significant difference compared to continuous infusion.^[8,17]^ In this study, there was no significant difference in the required additional analysis within 48 hours after surgery, and there was no significant difference in the total dose and frequency of bolus injection of local anesthetic into the epidural space, but there was a difference in PONV. When the PIEB technique is applied, the infusion rate is increased, and the drug spreads to high levels in the epidural space, causing hypotension, which seems to be the cause of PONV. In an article comparing the effects of PIEB technique during thoracic surgery, it was reported that hypotension was more common than the continuous infusion technique,^[8]^ and the incidence of hypotension was lower when the local anesthetic was slowly injected.^[18]^

As a limitation of this study, only the pain control effect of the PIEB injection was limited to improving sleep quality. It is considered that the effect on the improvement of the patient’s prognostic improvement will also be studied more diversely, and physiological functions such as postoperative lung capacity and respiratory function recovery, behavior, and activity conditions will be assessed extensively. In evaluating the patients’ sleep condition, the investigator obtained the results through a survey that relied on the patients’ subjective memory. In addition, our study lacked the number of samples, thus, it is necessary to study a larger sample. Some parameters such as night PCEA drug dose, frequency of bolus at night, and others are expected to be statically meaningful with large sample sizes. During the procedure, since the thoracic epidural catheter insertion is a procedure with large failure rates and adverse events, there is a need for a study that assesses the negative factors such as the successful post-procedural pain aspects, PONV, hypotension, and the procedure itself.

In conclusion, the PIEB injection of PCEA reduces postoperative pain and improves sleep quality in patients who underwent thoracotomy for lung cancer or other lung diseases. This reduction in pain and improvement in sleep quality are due to PIEB injection that automatically administer bolus even during sleep, reducing the frequency of waking up from complaining of pain during sleep.

## Author contributions

**Conceptualization:** Su-Sung Lee, Gyeong-Jo Byeon.

**Data curation:** Hye-Jin Kim

**Formal analysis:** Su-Sung Lee, Gyeong-Jo Byeon.

**Investigation:** Ji-Hye Baek, Soon-Ji Park

**Methodology:** Su-Sung Lee, Gyeong-Jo Byeon.

**Supervision:** Hee-Young Kim

**Writing—original draft:** Su-Sung Lee, Gyeong-Jo Byeon.

**Writing—review and editing:** Gyeong-Jo Byeon.
